# Heart Rate Variability: Marker of the Impact of Cardiovascular Disease on Intrinsic Capacity in Older Adults

**DOI:** 10.3390/jcm14092981

**Published:** 2025-04-25

**Authors:** Ana-Maria Turcu, Adina Carmen Ilie, Sabinne-Marie Albișteanu, Gabriela Grigoraș, Iulia-Daniela Lungu, Ramona Ștefăniu, Anca Iuliana Pîslaru, Ioana Dana Alexa

**Affiliations:** Department of Medical Specialities II, Grigore T Popa University of Medicine and Pharmacy, 700115 Iasi, Romania

**Keywords:** heart rate variability, old people, intrinsic capacity

## Abstract

**Objective:** This study investigates the association between heart rate variability parameters—particularly SDNN or SDANN—and components of intrinsic capacity in older adults, including functional, nutritional, cognitive, psycho-emotional domains, and frailty. Primary outcomes assess the relationship between SDNN and SDANN and frailty status and functional performance (ADL/IADL scores, handgrip strength). Secondary outcomes assess the relationship between SDNN and SDANN and cognitive status (MMSE), nutritional status (MNA, BMI, total protein, hemoglobin, visceral fat), emotional well-being (GDS), and urinary incontinence. **Methods:** This prospective exploratory study included 83 patients over the age of 65, hospitalized between January and October 2024. All participants underwent cardiovascular evaluation and evaluation of intrinsic capacity (frailty status, ADL/IADL, handgrip strength, MMSE, MNA, GDS, urinary incontinence). **Results**: Patients were grouped by SDNN values (<128 ms vs. ≥128 ms). Those with lower SDNN had significantly lower MNA scores (*p* = 0.047), lower hemoglobin (ρ = 0.220, *p* = 0.046), and higher GDS scores (ρ = −0.219, *p* = 0.047), indicating poorer nutritional and emotional status. SDANN was negatively correlated with frailty scores (ρ = −0.269, *p* = 0.014) and positively correlated with ADL scores (ρ = 0.247, *p* = 0.024), suggesting better functional independence. Handgrip strength was significantly predicted by both SDNN (*p* = 0.002) and SDANN (*p* = 0.002) in univariable linear regression. Visceral fat levels were positively correlated with SDNN (ρ = 0.292, *p* = 0.007), though BMI was not. No significant associations were found between HRV parameters and MMSE scores or urinary incontinence. **Conclusions**: HRV parameters, particularly SDNN and SDANN, show modest but significant associations with intrinsic capacity components such as frailty, functional performance, nutritional status, and emotional well-being in older adults. These findings suggest that SDNN and SDANN may serve as non-invasive markers for the early identification of declines in intrinsic capacity. Larger longitudinal studies are needed to validate these preliminary results.

## 1. Introduction

Heart rate variability (HRV) reflects the variation in the time between consecutive heartbeats and serves as a non-invasive marker of autonomic nervous system (ANS) activity. In older adults, age-related changes in sympathetic and parasympathetic tone can lead to autonomic imbalance and reduced HRV, which have been linked to increased cardiovascular risk, frailty, and functional decline.

SDNN (Standard Deviation of Normal-to-Normal Intervals) is a time-domain measure of heart rate variability (HRV). It represents the variability in the time intervals between successive normal heartbeats (R–R intervals) over a specific period, typically measured in milliseconds (ms). SDNN is one of the most widely used HRV parameters because it reflects the overall autonomic nervous system (ANS) function, particularly the balance between the sympathetic and parasympathetic branches [1,2,3]. A higher SDNN value suggests better autonomic nervous system regulation, implying a healthy balance between the sympathetic (fight or flight) and parasympathetic (rest and digest) branches. It is associated with good cardiovascular health, resilience to stress, and effective recovery [1,4]. In older adults, higher SDNN values are typically seen in individuals who are in better physical health and have a lower risk of developing cardiovascular diseases or other geriatric syndromes [5]. In contrast, lower SDNN values are linked to frailty, functional decline, and an increased risk of falls or disability. It also correlates with poorer outcomes in conditions like depression, neurodegenerative diseases, and other geriatric syndromes [6,7,8,9].

An additional and equally important parameter is SDANN (Standard Deviation of the Average NN intervals), which reflects long-term components of heart rate variability by representing the standard deviation of the average NN intervals calculated over successive 5 min segments. Higher levels are independently associated with better cognitive performance, suggesting a positive correlation between parasympathetic activity and cognitive function [10]. Other specific studies directly correlating SDANN with functional status, nutritional disorders, frailty, depression, and urinary incontinence are limited, and SDANN was not directly measured [11,12].

Studies evaluating HRV parameters in the elderly population are relatively few, and those that exist show associations between HRV and a wide range of age-related conditions, including neurocognitive disorders, depression, and functional decline [1,4,13].

Intrinsic capacity refers to the composite of an individual’s physical and mental abilities that enable them to perform activities of daily living and maintain autonomy in later life. In accordance with the WHO ICOPE framework, intrinsic capacity (IC) encompasses five key domains: cognition, mobility, psychological well-being, vitality/nutrition, and sensory function (vision and hearing) [13]. As cardiovascular diseases influence both physical and cognitive functions through autonomic dysfunction, HRV may represent an integrated marker of IC decline.

Cardiovascular disease is one of the leading causes of morbidity and mortality in older adults, often contributing to a progressive decline in physical function, frailty, and an increased risk of mortality. Since it can impair autonomic function and affect HRV, assessing HRV as an early marker may help prevent or mitigate its adverse effects in aging populations.

As HRV reflects the balance between sympathetic and parasympathetic activity, it serves as a sensitive marker of autonomic dysfunction, which is commonly seen in cardiovascular disease and aging. Low HRV has been linked to increased mortality, frailty, and functional decline in older adults. Therefore, HRV could potentially be used as a non-invasive tool to assess cardiovascular health and, by extension, the overall intrinsic capacity of elderly patients [14]. Old patients with a higher resting heart rate have poorer performance on both functional status scales, with lower scores on the ADL and IADL tests. Furthermore, SDNN correlated with lower scores on both functional tests, suggesting it could be considered a marker of physical performance [15]. Studies suggest a strong association between HRV dynamics and the presence of frailty [16,17].

Although HRV has been linked to individual geriatric syndromes—such as depression, frailty, and cognitive impairment—its broader potential as a marker of intrinsic capacity remains underexplored. Our study aims to explore these relationships in greater depth, with a particular focus on HRV parameters, such as SDNN, and their impact on the physical and functional health of older adults. Additionally, we aim to clarify how HRV can serve as an early marker for the decline of intrinsic capacity within the context of a comprehensive assessment that includes cognitive, nutritional, and emotional health. In this context, identifying a predictive model based on HRV could open new perspectives for the prevention and management of geriatric syndromes, thereby contributing to a personalized care plan that supports the independence of elderly patients [6,18,19].

## 2. Study Objective

The main objective is to investigate the relationship between HRV parameters and the components of intrinsic capacity (functional, nutritional, cognitive, psycho-emotional, and frailty) in older adults, considering that HRV is influenced both by cardiovascular status and by autonomic nervous system dysfunctions, which are common in geriatric pathology.

The primary outcomes of this study were the correlations between HRV parameters—particularly SDNN—and frailty status (assessed by the Fried phenotype) and functional performance (ADL/IADL scores and handgrip strength). Secondary outcomes included correlations between HRV parameters—particularly SDNN—and cognitive status (MMSE), nutritional status (MNA, BMI, total protein, hemoglobin, visceral fat, emotional well-being (GDS)), and urinary incontinence as intrinsic capacity components.

## 3. Materials and Methods

This is a prospective exploratory study presenting results from January 2024 to October 2024. It includes 83 patients over the age of 65 who were hospitalized in the Geriatrics and Gerontology Department of the “Dr. C.I. Parhon” Clinical Hospital in Iași, Romania. This study is still ongoing, and we want to extend the initial sample. After the completion of enrolment, we aim to publish the new results that will strengthen the preliminary findings and increase statistical veracity. All subjects involved in the study provided written informed consent voluntarily.

The patients included in the study presented the following characteristics: over 65 years of age, provided consent and agreed to be enrolled in the study, were able to maintain an upright posture or had a minimal level of functionality necessary for body composition assessment, and had been previously diagnosed by a specialist or were identified during hospitalization with at least one deficiency (neurocognitive disorder, nutritional disorder, emotional status alteration, functional disorder, pre-frailty or frailty status, urinary incontinence).

The exclusion criteria were as follows: patients under 65 years of age, lack of informed consent, inability to obtain clinical or biochemical data, bedridden patients or those unable to maintain an upright posture (as body composition could not be evaluated via bioimpedance in these cases), and patients with pacemakers (the determination of body composition was also impossible to perform in this case).

All enrolled patients underwent a comprehensive cardiovascular evaluation (ECG, Holter ECG, echocardiography) and evaluation of the intrinsic capacity (IC). In the present study, we assessed four of the five domains in the ICOPE (Integrated Care for Older People) using validated instruments: cognition (Mini-Mental State Examination—MMSE), mobility (Activities of Daily Living—ADL, Instrumental Activities of Daily Living—IADL, Fried frailty phenotype, and handgrip strength—using dynamometer), psychological well-being (Geriatric Depression Scale—GDS), and nutritional status (Mini Nutritional Assessment—MNA, BMI, visceral fat, serum proteins, and hemoglobin levels). Sensory capacities (vision and hearing) were not assessed in this cohort. All the above-mentioned data was stored in an Excel database.

The primary outcomes of this study were the correlations between HRV parameters—particularly SDNN—and frailty status (assessed by the Fried phenotype) and functional performance (ADL/IADL scores, handgrip strength). Secondary outcomes included correlations between HRV parameters—particularly SDNN—and cognitive status (MMSE), nutritional status (MNA, BMI, total protein, hemoglobin, visceral fat, emotional well-being (GDS)), and urinary incontinence as intrinsic capacity components.

The SDNN and SDANN parameters, both part of heart rate variability analysis, were extracted from 24 h Holter ECG monitoring. According to existing literature, SDNN and SDANN values below 50 ms are considered low and are associated with an increased risk of cognitive impairment, functional decline, nutritional deficits, emotional disturbances, frailty, and urinary incontinence. Values between 50 and 100 ms are generally regarded as normal, while values exceeding 100 ms are considered optimal for supporting successful aging.

For the primary outcomes, frailty was assessed using the Fried phenotype, which includes a 5-item test evaluating exhaustion, involuntary weight loss, gait speed, muscle strength, and decreased energy levels. The presence of each of these items was scored with one point. Considering the score obtained from the Fried frailty phenotype, a patient is classified as normal at a score of 0, a score of 1 and 2 indicate a pre-frail condition, and scores of 3, 4, or 5 indicate a frail status [20]. We used the Fried phenotype because it is considered superior to other frailty assessment tools by providing a standardized, clinically validated, and easy-to-apply method. It is significantly associated with major health risks such as disability, hospitalization, and mortality.

Functional performance was determined using the following: 1. ADL (activities of daily living) and IADL (instrumental activities of daily living). ADL (daily basic activities) has a maximum score of 6, meaning a fully independent person, and a score of 3 or lower classifies the patient as fully dependent. The IADL test in geriatric evaluation has 8 items. A maximum score of 8 means a fully independent person and a score of 3 or lower classifies the patient as fully dependent [21]. 2. Handgrip strength was determined using a dynamometer for both hands. Three successive measurements were performed, and the highest value was considered. The values obtained in kilograms were interpreted according to sex and age. For males aged 65–69 years, normal muscle strength ranged from 28.2 kg to 44.0 kg, while for those aged 70–99 years, it ranged from 21.3 kg to 35.1 kg. Values below the lower limit were considered low, while those above the upper limit were considered high. For females aged 65–69 years, normal muscle strength ranged from 15.4 kg to 27.2 kg, while for those aged 70–99 years, it ranged from 14.7 kg to 24.5 kg. Values below the lower limit were considered low, while those above the upper limit were considered high [22].

For the secondary outcomes, the cognitive status was evaluated using the Mini-Mental State Examination (MMSE) scale. A maximum score is 30 points, and a minimum is 0 points. Normal cognitive function is considered for scores between 25 and 30 points, mild impairment for scores between 20 and 24, moderate impairment for scores between 10 and 19, and severe impairment for scores below 10 [23].

Nutritional status was assessed based on the following:

1. The Mini Nutritional Assessment (MNA) questionnaire, with scores between 24 and 30 indicating normal nutritional status (absence of malnutrition), scores between 17 and 23.5 indicating a risk of malnutrition, and scores below 17 indicating malnutrition [24].
2. Body Mass Index (BMI)—calculated using weight and height, with values under 18.5 kg/m^2^ indicating underweight, between 18.5 kg/m^2^ and 24.9 kg/m^2^ indicating normal weight, between 25 kg/m^2^ and 29.9 kg/m^2^ indicating overweight, 30–34.9 kg/m^2^ indicating class I obesity, 35–39.9 kg/m^2^ indicating class II obesity, and values above 40 kg/m^2^ indicating morbid obesity.
3. Lab values were collected from recent medical records (recent data—up to 7 days old). The lab test used: total protein levels were considered normal between 66 and 87 g/L, and the hemoglobin levels were considered normal between 11.5 and 15.7 g/dL. Values below the lower limit were considered low, while those above the upper limit were considered high.
4. Visceral fat. Determined using the Tanita BC-545N weighing scale, which is based on bioelectrical impedance analysis. This method requires the patient to stand upright, and the results are obtained after a single measurement. The device classifies the obtained values as normal/low/high based on age, sex, height, and the patient’s profile (sedentary or athletic). Values between 1 and 12 are considered normal, between 13 and 19 are elevated, and above 19 are very elevated.

Emotional well-being status was assessed using the Geriatric Depression Scale 15 items (GDS). A normal score is considered below 5, where a score between 5 and 8 indicates mild depression, 9–11 indicates moderate depression, and a score above 11 indicates severe depression [25].

Urinary incontinence was obtained from the patient and recorded as yes (presence) or no (absence).

This study did not include adjustments for comorbidities, lifestyle factors, or medication use. The patients included in the study frequently presented cardiovascular conditions (hypertension, heart failure, and even arrhythmias), neurological conditions (history of stroke), or rheumatological conditions (osteoporosis, gonarthrosis, coxarthrosis). This study provides partial data on the presence of atrial fibrillation in the studied group because it is considered an independent predictor regarding HRV parameters.

## 4. Statistical Analysis

All data were analyzed using IBM SPSS Statistics 25. Quantitative variables were tested for normality using the Shapiro–Wilk Test and were expressed as means with standard deviations or medians with interquartile ranges (See the Supplementary Data—Table S1). Quantitative independent variables with non-parametric distribution were tested between groups using the Mann–Whitney U Test. Quantitative independent variables with normal distribution were tested between groups using Student’s *t*-Test (after testing for equality of variances between groups according to Levene’s Test).

For quantitative independent variables with normal distribution, Student’s *t*-Test (after checking for homogeneity of variances between groups using Levene’s Test) was used, and correlations were quantified using Pearson’s correlation coefficients. Linear regression univariable and multivariable models were used for the prediction of the dependent quantitative variables analyzed in the study (to which most of the correlations were tested). Because of the multi-correlated nature of the HRV parameters, forward stepwise models were used to determine the best prediction observed in the univariable linear regression models, in which only univariable linear regression models were selected with the best prediction from independent HRV parameters. Afterwards, the HRV parameters’ prediction was adjusted with gender in multivariable models.

Multiple correlations were tested, using Pearson correlations for variables with a normal distribution, which was confirmed by the Shapiro–Wilk test. However, the manuscript data mainly includes quantitative variables with a non-parametric distribution, and correlations were assessed using Spearman’s rho coefficients.

Both simple and multiple correlations were performed, and for group comparison, participants were divided into two categories: those with SDNN below 128 and those with SDNN above 128. Statistical significance was considered at *p* < 0.05.

Models were tested for significance, normality of residuals, homoscedasticity, and multicollinearity. The measure of prediction was calculated as a beta coefficient with 95% confidence intervals along with significance values.

## 5. Results

### 5.1. Demographic Data

The patients included in the study had an average age of 75.64 ± 6.64 years, with a median age of 75. The majority of patients were female (63.9%). Age distribution showed that most patients were between 65 and 74 years old (48.2%), classified as young old, followed by 75–84 years old (39.8%), categorized as adult old, and 12% were over 85 years old (Table 1)

It was found that patients with lower SDNN values were younger (74 (68–78.5)), with *p* = 0.038. There were more women, although the difference was not statistically significant (*p* = 0.255), and patients aged between 65 and 75 predominated in both categories. (See the Supplementary Data—Table S2).

### 5.2. Primary Outcomes—Frailty Status and Functional Performance

There are no statistically significant differences between the two groups in our study sample (Table 1). We can observe that handgrip strength, as an expression of functional performance, is higher in the group with higher SDNN values, 20.76 ± 9.75 vs. 22.63 ± 9.09, even though it did not reach statistical significance (*p* = 0.370 vs. *p* = 0.435). In the univariable analysis, muscle strength in the right arm was significantly correlated with the following parameters: SDNN (*p* = 0.039, ρ = 0.227) and SDANN (*p* = 0.010, ρ = 0.283).

These were low positive correlations, indicating that patients with higher handgrip strength values were significantly more associated with higher values of SDNN and SDANN.

Linear regression univariable models using handgrip strength in the right arm as a dependent variable show a significant prediction of SDNN (B = 0.050731 (95% C.I.: 0.020014–0.081448), *p* = 0.002) and SDANN (B = 0.027155 (95% C.I.: 0.010034–0.044275), *p* = 0.002). The multivariable model was not valid due to multicollinearity.

SDNN itself did not demonstrate a direct statistically significant correlation with frailty scores, and its related parameter, SDANN (Standard Deviation of the Average NN intervals), was negatively and significantly correlated (ρ = −0.269, *p* = 0.014). This suggests that reduced long-term HRV is associated with greater frailty in older adults. Linear regression univariable models using the frailty score as a dependent variable show a significant prediction of SDANN (B = −0.002754 (95% C.I.: −0.004952–−0.000556), *p* = 0.015).

The multivariable model was not valid due to multicollinearity. The evaluation of functional status using the Activities of Daily Living (ADL) scale revealed a statistically significant positive correlation with SDANN (ρ = 0.247, *p* = 0.024). This finding indicates that individuals with greater functional independence tend to exhibit more favorable autonomic regulation, as reflected by higher SDANN values.

ADL was significantly correlated with SDANN (*p* = 0.024, ρ = 0.247), showing low positive correlations. This indicates that patients with higher ADL scores were significantly more associated with higher values of the SDANN. However, linear regression univariable models did not show positive correlations. IADL was not significantly correlated with SDNN or SDANN in univariate or multivariate analysis; data concordant with results presented in Table 2.

### 5.3. Secondary Outcomes

We did not find statistically significant differences in cognitive status, nutritional status, emotional well-being, or urinary incontinence between older patients with low versus high SDNN values. However, the group with lower SDNN showed more affected emotional status (GDS = 6 (4–9) vs. 5 (3–8)) and impaired nutritional status (with lower values of MNA, BMI, hemoglobin, and total fat), even though these differences did not reach statistical significance. Patients with higher SDNN values had a greater proportion of high and very high levels of visceral fat compared to those with lower SDNN values, who exhibited a higher percentage of normal visceral fat (*p* = 0.037).

Cognitive status was assessed using the Mini-Mental State Examination (MMSE), a standardized tool widely employed in geriatric evaluations. Among the studied cohort, 80.7% (n = 67) of patients showed no signs of cognitive impairment, 12% (n = 10) were classified with mild cognitive impairment, and 7.2% (n = 6) with moderate impairment. Upon analysis, no statistically significant associations were observed between MMSE scores and SDANN or SDNN (*p* > 0.05).

SDNN values did not vary meaningfully across cognitive status groups, and cognitive impairment was not considered a confounding factor in subsequent analyses. The SDNN parameter demonstrated a statistically significant positive correlation with nutritional status as assessed by the Mini Nutritional Assessment (MNA) (ρ = 0.219, *p* = 0.047). This suggests that older adults with better nutritional profiles tend to exhibit higher SDNN values, indicating a more favorable autonomic balance. Moreover, in univariable linear regression analysis, SDNN was a significant predictor of MNA scores (B = 0.014974, 95% CI: 0.002578–0.027369, *p* = 0.019), reinforcing the link between improved heart rate variability and better nutritional status. In the stepwise forward regression model, SDNN remained the independent predictor of nutritional status.

Moreover, no significant correlation was found between Body Mass Index (BMI) and the SDNN parameter. SDNN was significantly and positively correlated with hemoglobin levels (*p* = 0.046, ρ = 0.220), indicating that individuals with higher hemoglobin values tend to exhibit increased autonomic function, as reflected by higher SDNN values. However, in the univariable linear regression analysis, hemoglobin did not emerge as a statistically significant predictor of SDNN (*p* = 0.067), suggesting that while an association exists, it may not be strong enough to support a predictive relationship in this model. Total serum protein levels were significantly and positively correlated with SDANN (*p* = 0.046, ρ = 0.221). This low-grade correlation suggests that higher protein levels, potentially reflecting better nutritional status, are associated with more stable autonomic regulation over longer periods. Visceral fat exhibited a low positive correlation with SDNN (*p* = 0.007, ρ = 0.292), suggesting that individuals with higher levels of visceral adiposity tend to have increased autonomic modulation, as reflected by SDNN. Furthermore, univariable linear regression demonstrated that SDNN significantly predicted visceral fat levels (B = 0.019313; 95% CI: 0.003389–0.035237; *p* = 0.018).

In Table 3, it can be observed that patients with lower SDNN values present higher GDS scores (6 (4–9), indicating mild depression) compared to patients with higher SDNN values, who had a GDS of 5 (3–9), indicating no depression. However, the difference did not reach statistical significance.

Depressive symptoms, evaluated using the Geriatric Depression Scale (GDS), showed significant associations with heart rate variability parameters. Specifically, SDNN exhibited a statistically significant low negative correlation with GDS scores (*p* = 0.047, ρ = −0.219), indicating that higher levels of depressive symptomatology were modestly associated with reduced autonomic function, as reflected by lower SDNN. Moreover, univariable linear regression analysis identified SDNN as a significant negative predictor of GDS scores (B = −0.013657; 95% CI: –0.024875 to −0.002439; *p* = 0.018). No significant associations between SDANN and GDS were reported in the available data. The multivariable model was not valid due to multicollinearity.

In Table 3, the data showed that patients with SDNN lower had urinary incontinence compared to those with higher SDNN values, who showed a lower prevalence of incontinence, although the difference did not reach statistical significance (*p* = 0.238). No statistically significant associations were found between SDNN or SDANN and urinary incontinence in the data analyzed.

## 6. Discussion

This study aimed to evaluate the importance of SDNN and SDANN, the most representative time-domain parameters of heart rate variability in relation to key components of intrinsic capacity in the senior population of Iasi, Romania. This represents the first prospective study of its kind conducted in Romania, currently in progress, with preliminary findings presented herein. Future publications will report on the extrapolated and comprehensive results.

The existing literature indicates that the direct relationship between SDNN or SDANN and components of ICOPE is underexplored.

Analyses revealed gender differences in heart rate variability (HRV) parameters. Female patients showed more significant reductions in SDNN compared to male patients. This finding is confirmed in the study published in 2020, which included 1287 patients. Overall, men tend to exhibit higher HRV values, which could be attributed to testosterone’s role in enhancing sympathetic nervous system activity, whereas women with greater parasympathetic activity tend to have lower HRV [26,27].

In our study, the primary outcomes did not demonstrate a direct, statistically significant correlation between SDNN and frailty scores, but sustained a negative and significant correlation with SDANN.

The literature data support that no direct data studies prove the relationship between SDNN or SDANN parameters and frailty. However, a pilot study that aimed to evaluate autonomic cardiac modulation in non-frail, pre-frail, and frail status patients supports the idea that there are no significant differences between SDNN parameters and the non-frail, pre-frail, and frail groups in the lying position [15,28]. Additionally, another article suggests that there are no significant differences regarding SDNN or SDANN based on posture between non-frail and pre-frail individuals [2].

Another recent study, published in 2024, contradicts the previous findings, stating that time-domain parameters, represented by SDNN, were significantly lower in frail subjects compared to the other groups [29]. So, more studies are necessary to sustain the previous data. Our article aims to fill that gap.

Our results showed that the ADL scale revealed a statistically significant positive correlation with SDANN but not with IADL. The PROSPER study, the most representative one in the old population, supports the fact that a decrease in the SDNN parameter was associated with an increased risk of developing functional dependence [15,30]. Also, another study supports that the higher resting heart rate and a lower SDNN remained significantly associated with a higher risk of decline in both ADL and IADL in the fully adjusted model [15]. We consider that our partial result is encompassed within the previously discussed findings; however, to more accurately define and validate the association with SDNN, further research involving a larger study population and adjusted statistical analyses is warranted.

The evaluation of handgrip strength and its relationship, part of the functional status assessment, with SDNN or SDANN parameters shows that increased handgrip strength correlates with higher values of SDNN and SDANN as markers in the autonomic nervous system. These findings are also supported by the literature, as described in an article on a population with low cardiovascular risk [31]. A recent study published in 2024, which includes 132 old hospitalized patients, demonstrated that higher SDNN values are associated with strong muscle strength, which can be a negative factor in the occurrence of sarcopenia [32]. Another research suggests that isometric handgrip training does not have a significant effect on SDNN or SDANN [33].

The secondary outcomes suggest that there are no established associations between SDNN or SDANN and neurocognitive function. These findings are contradicted by a cross-sectional and longitudinal study conducted between 2012 and 2018, which included 8,507 participants and aimed to assess neurocognitive function based on cardiovascular evaluation. Cognitive performance was measured using both diagnostic tools, the Mini-Mental State Examination (MMSE) and the Montreal Cognitive Assessment (MOCA). The results support the findings that higher SDNN values were associated with better cognitive performance in longitudinal assessments, and this association was observed across both diagnostic scales [34]. Our result is interpreted in the context that the patients in our study group had normal MMSE values (27 (25–29)). Further research is required for statistically significant data and strong conclusions.

The SDNN parameter demonstrated a statistically significant positive correlation with nutritional status, as assessed by the Mini Nutritional Assessment. After the adjustments, SDNN remained the independent predictor of nutritional status but without statistical power. Studies on the interaction between HRV and nutritional status are limited, with more focus on obesity and preventive measures than malnutrition or malnutrition risk. In our study we find no statistically significant correlation between Body Mass Index (BMI) and the SDNN parameter. This is considered secondary to the fact that the patients included in the study have an overweight status, both in the group with decreased and increased SDNN.

Research suggests that a healthy diet can increase SDNN. A study conducted on a sample of overweight postmenopausal women demonstrated that a moderate weight loss of approximately 3.9 kg was associated with a reduction in heart rate and a significant increase in SDNN, a key marker of autonomic nervous system activity [35]. In contrast, another study investigating the effects of a hypercaloric diet in a population of healthy volunteers reported that an induced weight gain of approximately 4 kg over eight weeks was not directly associated with changes in SDNN values [36]. The discrepancy between these findings suggests the presence of confounding factors or methodological differences across studies, emphasizing the need for further research to elucidate the relationship between body weight changes and heart rate variability.

The findings in our study suggest that individuals with better SDNN values had high and very high percentages of visceral fat compared with those with lower SDNN values. A 2024 study including healthy adult males in Saudi Arabia reported a positive correlation between lower visceral fat and higher SDNN values [37]. This aspect is further supported by recent evidence suggesting that the presence of epicardial adipose tissue is associated with type 2 diabetes mellitus and a reduction in SDNN [38]. Similarly, abdominal adiposity has also been linked to decreased SDNN values [39]. We interpret our results as being influenced by the limited sample size, the presence of multiple comorbidities, and the specific lifestyle characteristics of the study population. Therefore, we emphasize the need for further investigations involving a larger and more homogeneous cohort to validate these findings.

Total serum protein levels were significantly and positively correlated with SDANN, but in our group comparison, there are no statistically significant differences between patients with low vs. high SDNN values. The literature data suggest that nutritional biomarkers, particularly those related to protein status, may influence cardiac autonomic function, as reflected by HRV parameters like SDNN. Data from the literature confirm, through a study conducted on 426 patients with subacute stroke, that deficiencies in serum levels of albumin were significantly associated with lower HRV parameters, including SDNN [40]. However, the direct impact of serum total protein levels on SDNN remains to be elucidated, and further research is needed to explore the specific role of total protein levels in HRV and their potential implications for cardiovascular health.

Our findings suggest that there was a statistically significant positive correlation between serum hemoglobin levels and SDNN, with no significant differences between the SDNN groups. This fact indicated that elevated hemoglobin levels may be associated with improved autonomic cardiovascular regulation, as reflected by higher SDNN values. The studies confirm that lower hemoglobin levels are generally associated with higher HRV, potentially reflecting increased parasympathetic activity [41].

Our findings indicate that higher scores on the Geriatric Depression Scale are modestly associated with lower SDNN values, suggesting a potential link between depressive symptoms and diminished autonomic regulation. This observation is consistent with the evidence reported in the literature. A cross-sectional study involving 189 patients with stable coronary artery disease demonstrated, through multiple linear regression analysis, that depressive symptoms were significantly associated with reduced HRV, particularly lower SDNN and SDANN values [42]. Furthermore, a meta-analysis encompassing 11 studies investigating HRV in older adults with depression revealed a significant reduction in HRV parameters among depressed individuals compared to healthy controls, with SDNN and SDANN specifically identified as markedly decreased in the depressive cohort [43]. However, a larger patient cohort is required to strengthen our findings. Additionally, further studies are needed to elucidate the underlying mechanisms involved.

Our findings did not demonstrate significant differences in SDNN or SDANN values between patients with urinary incontinence. However, a higher proportion of patients with urinary incontinence exhibited lower SDNN values (36.6% vs. 23.8%), suggesting a potential link between autonomic dysfunction and urinary incontinence (UI). This observation is consistent with previous research indicating that changes in HRV, specifically SDNN and SDANN, may be associated with different types of urinary incontinence, including overactive bladder and stress urinary incontinence [44]. Several studies have shown that alterations in autonomic nervous system function, reflected through changes in HRV parameters, are common in patients with urinary incontinence. For instance, patients with overactive bladder syndrome often present with an imbalance between sympathetic and parasympathetic activity, which can be observed through HRV indices such as SDNN and SDANN [45]. This aspect highlights the complex interplay between the autonomic nervous system and bladder function, suggesting that HRV could serve as an important marker for evaluating autonomic dysfunction in individuals with UI.

Despite these promising observations, the results of the present study are not definitive, and further research with larger, more homogeneous cohorts is required to fully understand the relationship between HRV parameters and urinary incontinence. Additionally, further studies should explore the potential impact of other variables, such as age, comorbidities, and medication use, on these associations. Overall, this study contributes to the growing body of evidence suggesting that HRV, including SDNN and SDANN, may be useful in assessing the autonomic dysregulation associated with urinary incontinence. Future research may help clarify the potential clinical applications of HRV monitoring in managing UI and related disorders.

## 7. Study Limitations

The main limitations of this study include the small sample size, which limits statistical power and the reliability of observed associations. Moreover, the presence of multiple comorbidities, heterogeneous lifestyle factors, and varied medication use among participants may have confounded HRV measurements, particularly SDNN and SDANN. Additionally, as this study was conducted within a single-center setting, the generalizability of the findings to the wider geriatric population remains limited.

Future research is planned to include a larger cohort and to control the confounding variables discussed above. Nonetheless, this study is among the few that directly examines the relationship between key time-domain HRV parameters—SDNN and SDANN—and core ICOPE components.

## 8. Conclusions

SDNN and SDANN, as the most representative parameters of heart rate variability, suggest a potential association with ICOPE components. So, determining SDNN and SDANN may be a useful tool in determining the intrinsic capacity of the following components: functional status, emotional well-being, nutritional status, cognitive status, and frailty.

Evaluating the intrinsic capacity of old patients requires numerous parameters, especially in determining health status and well-being, and SDNN and SDANN may be some of those.

Alterations in SDNN and SDANN parameters may be in relation to alterations of the intrinsic capacity, especially emotional well-being (depressive disorders), nutritional status (with its numerous parameters), and functional performance (handgrip strength).

However, further research is needed to confirm and validate these preliminary findings.

## Figures and Tables

**Table 1:** Demographic characteristics of the analysed patients depending on the SDNN values (below vs. above the median).

Parameter (Mean ± SD, Median (IQR)/Nr., %)	Total (N = 83)	SDNN < 128 (N= 41)	SDNN ≥ 128 (N = 42)	*p*
**Age**	75.64 ± 6.64, 75 (71–80)	74 (68–78.5)	76.5 (72–82)	0.038 *
**Gender (male)**	30 (36.1%)	12 (29.3%)	18 (42.9%)	0.255 **
**Gender** **(female)**	53 (63.9%)	29 (70.7%)	24 (57.1%)	
**Age category**				0.479 **
**65–74 years**	40 (48.2%)	21 (51.2%)	19 (45.2%)	
***Female***	24 (60%)	14 (66.7%)	10 (52.6%)	0.520 **
***Male***	16 (40%)	7 (33.3%)	9 (47.4%)	
**75–84 years**	33 (39.8%)	17 (41.5%)	16 (38.1%)	
***Female***	22 (66.7%)	13 (76.5%)	9 (56.3%)	0.282 **
***Male***	11 (33.3%)	4 (23.5%)	7 (43.8%)	
**≥85 years**	10 (12%)	3 (7.3%)	7 (16.7%)	
***Female***	7 (70%)	2 (66.7%)	5 (71.4%)	1.000 **
***Male***	3 (30%)	1 (33.3%)	2 (28.6%)	

* Mann–Whitney U Test, ** Fisher’s Exact Test. SD—Standard Deviation, IQR—Interquartile Range, N—Number, SDNN—Standard Deviation of Normal-to Normal Intervals.

**Table 2:** Frailty status and functional performance depending on SDNN values (below vs. above the median).

Parameter (Mean ± SD, Median (IQR)/Nr., %)	Total (N = 83)	SDNN < 128 (N = 41)	SDNN ≥ 128 (N = 42)	*p*
**Fried phenotype score**	3 (2–4)	3 (2–4)	3 (2–3.25)	0.310 *
**ADL**	6 (5–6)	6 (5–6)	6 (5–6)	0.975 *
**IADL**	7 (6–8)	7 (6–8)	7 (5–8)	0.421 *
**Handgrip strength—right arm** **(Mean ± SD)**	21.7 ± 9.41, 20.8 (14.1–27.8)	20.76 ± 9.75	22.63 ± 9.09	0.370 ***
**Handgrip strength—right arm (category)**				
***Low***	24 (28.9%)	11 (26.8%)	13 (31%)	0.435 **
***Normal***	52 (62.7%)	28 (68.3%)	24 (57.1%)	
***High***	7 (8.4%)	2 (4.9%)	5 (11.9%)	
**Handgrip strength—left arm**	20.87 ± 8.85, 19.8 (14.4–26.9)	20.41 ± 9	21.33 ± 8.78	0.639 ***
**Handgrip strength—left arm (category)**				
***Low***	26 (31.3%)	12 (29.3%)	14 (33.3%)	0.612 *
***Normal***	51 (61.4%)	27 (65.9%)	24 (57.1%)	
***High***	6 (7.2%)	2 (4.9%)	4 (9.5%)	

* Mann–Whitney U Test, ** Fisher’s Exact Test, *** Student *t*-Test. SD—Standard Deviation, IQR—Interquartile Range, N—Number, SDNN—Standard Deviation of Normal-to Normal Intervals, ADL-Activities of Daily Living, IADL – instrumental Activities of Daily Living

**Table 3:** Secondary outcomes depending on SDNN values (below vs. above the median).

Parameter (Mean ± SD, Median (IQR)/Nr., %)		Total (N = 83)	SDNN < 128 (N = 41)	SDNN ≥ 128 (N = 42)	*p*
**MMSE**		27 (25–29)	28 (24–29)	27 (26–29)	0.741 *
**GDS**		5 (3–9)	6 (4–9)	5 (3–8)	0.161 *
**Nutritional status**	**MNA**	24 (21–26)	24 (20.25–26)	25 (22–26.25)	0.176 *
	**BMI (kg/m^2^)**	28.97 ± 5.72, 28.3 (25.9–31.9)	28.57 ± 6.28	29.37 ± 5.17	0.526 ***
	**Total proteins (g/L)**	72.27 ± 5.17	72.02 ± 4.57	72.51 ± 5.75	0.672 ***
	**Hemoglobin (g/dL)**	13.3 (12.4–14.4)	13.3 (12.1–14.2)	13.5 (12.6–15.1)	0.266 *
	**Visceral fat (Mean ± SD) (% Total Weight)**	13.56 ± 4.74	12.61 ± 4.75	14.49 ± 4.6	0.071 ***
	**Visceral fat (Nr., %)**				
	***Normal***	28 (33.7%)	19 (46.3%)	9 (21.4%)	0.037 **
	***High***	39 (47%)	17 (41.5%)	22 (52.4%)	
	***Very high***	16 (19.3%)	5 (12.2%)	11 (26.2%)	
**Urinary incontinence**		25 (30.1%)	15 (36.6%)	10 (23.8%)	0.238 **

* Mann–Whitney U Test, ** Fisher’s Exact Test, *** Student *t*-Test. SD—Standard Deviation, IQR—Interquartile Range, N/Nr—Number, SDNN—Standard Deviation of Normal-to-Normal Intervals, MMSE—Mini Mental State Examination, GDS—Geriatric Depression Scale, BMI—Body Mass Index, MNA—Mini Nutritional Assessment.

## Data Availability

The original contributions presented in this study are included in the article/Supplementary Material. Further inquiries can be directed to the corresponding author.

## Supplementary Materials

The following supporting information can be downloaded at https://www.mdpi.com/article/10.3390/jcm14092981/s1, Table S1: Normality testing of the included parameters; Table S2: Multivariable linear regression models used in the prediction of analysed parameters in the study.
