# Supplementary material for: Heart Rate Variability: Marker of the Impact of Cardiovascular Disease on Intrinsic Capacity in Older Adults
Source: J Clin Med. 2025 Apr 25;14(9):2981. doi: 10.3390/jcm14092981 (PMC12072958; doi:10.3390/jcm14092981)
Supplement: Supplementary file 1 [file jcm-14-02981-s001.zip › jcm-3496101-supplementary.pdf]

Table S1. Normality testing of the included parameters

Parameter	*p-value (Shapiro-Wilk Test)*
MNA	***0.001***
Variability	***<0.001***
SDNN	***<0.001***
SDANN	***<0.001***
ULF	***<0.001***
Bone mass	***<0.001***
LF	***<0.001***
HF	***<0.001***
BMI	*0.100*
Visceral fat	*0.258*
SDNN-ix	***<0.001***
RMSSD	***<0.001***
p50NN	***<0.001***
VLF	***<0.001***
nLF	***<0.001***
LF/HF	***<0.001***
Muscle strength – right arm	*0.280*
Muscle strength – right arm	***0.035***
Total protein	*0.325*
Hemoglobin	***0.008***
ADL	***<0.001***
IADL	***<0.001***
Number of medications – discharge	***0.004***
TINN	*0.159*
GDS	***0.005***
Freid phenotype score	***<0.001***

Table S2. Multivariable linear regression models used in the prediction of analyzed parameters in the study

D.V. = MNA/I.V.	**Univariable**		**Multivariable**	
	**Beta (95% C.I.)**	**p**	**Beta (95% C.I.)**	**p**
**SDNN**	0.015 (0.003-0.027)	**0.019**	0.011 (-0.002 – 0.024)	0.091
**Gender (Female)**	-2.062 (-3.689 - -0.435)	**0.014**	-1.594 (-3.291 – 0.104)	0.065
**Atrial fibrillation**	0.687 (-0.974 – 2.347)	0.413	-	-
D.V. = Bone mass/I.V.	**Beta (95% C.I.)**	**p**	**Beta (95% C.I.)**	**p**

D.V. = Dependent variable, I.V. = Independent variable, SDNN= Standard Deviation of Normal-to Normal Intervals**, MNA=**Mini Nutritional Assessment
